# Healthy lifestyle behaviours are positively and independently associated with academic achievement: An analysis of self-reported data from a nationally representative sample of Canadian early adolescents

**DOI:** 10.1371/journal.pone.0181938

**Published:** 2017-07-28

**Authors:** Erin L. Faught, Doug Gleddie, Kate E. Storey, Colleen M. Davison, Paul J. Veugelers

**Affiliations:** 1 School of Public Health, University of Alberta, Edmonton, Alberta, Canada; 2 Department of Elementary Education, University of Alberta, Edmonton, Alberta, Canada; 3 Departments of Public Health Sciences and Emergency Medicine, Queens University, Kingston, Ontario, Canada; McMaster University, CANADA

## Abstract

**Introduction:**

The lifestyle behaviours of early adolescents, including diet, physical activity, sleep, and screen usage, are well established contributors to health. These behaviours have also been shown to be associated with academic achievement. Poor academic achievement can additionally contribute to poorer health over the lifespan. This study aims to characterize the associations between health behaviours and self-reported academic achievement.

**Methods:**

Data from the 2014 Canadian Health Behaviour in School-Aged Children Study (n = 28,608, ages 11–15) were analyzed. Students provided self-report of academic achievement, diet, physical activity, sleep duration, recreational screen time usage, height, weight, and socioeconomic status. Multi-level logistic regression was used to assess the relationship of lifestyle behaviours and body weight status with academic achievement while considering sex, age, and socioeconomic status as potential confounders.

**Results:**

All health behaviours exhibited independent associations with academic achievement. Frequent consumption of vegetables and fruits, breakfast and dinner with family and regular physical activity were positively associated with higher levels of academic achievement, while frequent consumption of junk food, not meeting sleep recommendations, and overweight and obesity were negatively associated with high academic achievement.

**Conclusions:**

The present findings demonstrate that lifestyle behaviours are associated with academic achievement, potentially identifying these lifestyle behaviours as effective targets to improve academic achievement in early adolescents. These findings also justify investments in school-based health promotion initiatives.

## Introduction

The physical activity, diet, sleep, and screen time of children and youth are an important concern both to public health professionals and to society today. Consistently, population-level evaluations demonstrate that both children and adolescents in Canada are failing to achieve established healthful recommendations for all of these behaviours [1–3]. Consequently, children and adolescents are experiencing adverse health consequences at unprecedented rates, including obesity [4] and type 2 diabetes [5], putting them at risk for ill health and chronic disease across their lifespan.

In addition to consequences to health, these behaviours (diet, physical activity, sleep, and screen time) have been shown to be associated with children and adolescents’ academic achievement. Reaching optimal nutrition, physical activity, and sleep levels have demonstrated importance for academic achievement [6–8] while excess recreational screen time has been shown to negatively influence academic achievement [9]. In addition, overweight and obesity have been associated with poorer academic achievement [10], although recent reviews have noted that studies including potential mediators and moderators of this relationship, such as physical activity, diet, sleep, screen time, and socioeconomic status, have rarely been considered in these analyses [11,12]. The high prevalence of unhealthy behaviours and their negative associations with academic achievement is concerning for children and adolescents as school engagement and education are demonstrated protective factors against the development of adverse health over the entire lifespan [13,14]. Thus, these unhealthy behaviours contribute to adverse health consequences through direct physiological effects and by negatively influencing the likelihood of succeeding in school, resulting in lower socioeconomic status later in life [15]. The health-education relationship also suggests that effective interventions which improve the physical activity levels, diets, sleep, and screen use of youth, such as school-based health promotion, can have direct benefits on health as well as improve educational attainment, resulting in a healthier, more prosperous, and productive next generation [16]. Several school-based health promotion programs have been shown to achieve demonstrated improvements in lifestyle behaviours and academic achievement, though studies assessing this are few and further evidence is needed [17, 18].

Although each of diet, physical activity, sleep, screen time, and body weight status have established relationships with academic achievement, few studies [19–22] have considered all of these health behaviours simultaneously in an analysis to determine their independent effects on academic achievement. Findings from these limited studies indicate that exhibiting a healthy diet, adequate physical activity and sleep, and reduced screen time, have individual, positive associations with academic achievement regardless of body weight status with the exception of one study [19–22]. This evidence is supportive of school health approaches that are multi-componential, as focusing on singular behaviours may not have as substantial as an effect on academic achievement as would considering multiple healthy behaviours. In addition, this evidence supports the idea that health promotion to improve physical activity, diet is beneficial for the academic achievement all students, not simply those who are overweight or obese.

Our objective is to complement and expand on the limited existing studies that have aimed to investigate the independent associations of physical activity, diet, sleep, screen time, and body weight status on academic achievement using a large, population-based sample of early adolescents (age 11–15) from all provinces in Canada. This is the largest study to date and the first to use a representative sample of early adolescents in Canada. These findings can be used to inform population-level interventions to improve the physical activity, diet, sleep, and screen use of children and youth and consequently reduce their likelihood of adverse health and academic achievement outcomes.

## Methods

This work is a secondary data analysis of data from the Canadian version (Cycle 7) of the 2014 Health Behaviour in School-aged Children (HBSC) study [23]. This questionnaire is conducted in collaboration with the World Health Organization (WHO) [23]. Data was requested for this secondary analysis using a standard procedure that can be found at http://www.uib.no/en/hbscdata/94226/access-other-hbsc-survey-data. In Canada, consistent with all participating countries, this survey was carried out among a representative sample of Grade 6–10 (focused on 11–15 year old) students in all 13 provinces and territories excluding students who were on First Nation or Indian reserves, private and home schooled students, youth not in a school setting, or incarcerated youth [24]. All provinces and territories invited to participate in the survey consented to participation. A two-stage cluster sampling approach was used in most provinces: school jurisdictions were identified and categorized based on key characteristics (language of instruction, public/separate designation, and size of community). Upon jurisdictional consent, schools were randomly selected to participate within each jurisdiction. Schools had the opportunity to decide if the survey would be completed online or using paper and pen. Surveys were administered during school time (45–70 minute single session) and overseen by a teacher. Surveys asked a wide variety of questions about health and lifestyle behaviours as well as socio-demographic information. Further information about the HBSC survey can be found at http://healthycanadians.gc.ca/publications/science-research-sciences-recherches/health-behaviour-children-canada-2015-comportements-sante-jeunes/index-eng.php#c1a2. There was a 77% student response rate for the HBSC survey, resulting in 29,837 student participants [24]. After excluding students without complete information for academic achievement, 28,608 (96%) students were considered in the analysis. Sampling weights were applied to the sample in order to achieve representativeness of Canadian youth by grade, gender and province or territory. Ethical approval for the HBSC study in Canada was obtained from the Queen’s University General Research Ethics Board (Approval GMISC-062-13) and from Health Canada and the Public Health Agency of Canada.

### Academic achievement

Students self-reported their academic achievement by responding to the following question: ‘Which of the following best describes your marks during the past year?’ Possible responses were: ‘Mostly A’s/above 85%/or level 4’, ‘Mostly A’s and B’s/between 70 and 84%/or level 3 and 4’, ‘Mostly B’s and C’s/between 60 and 69%/or level 3’, ‘Mostly C’s/between 50 and 59%/ or level 2’, and ‘Mostly letter grades below C/below 50%/or level 1’. For ease of readibility, these categories are henceforth referred to by their letter categories. These categories were collapsed into two categories: ‘Excellent’ (Mostly A’s, Mostly A’s and B’s) and Fair (Mostly B’s and C’s and below).

### Physical activity

Physical activity was assessed using the question: “Over a typical or usual week, on how many days are you physically active for a total of at least 60 minutes per day?” Possible responses were 0, 1, 2, 3, 4, 5, 6, or 7 days per week. This question corresponds with the Canadian 24-hour Movement Guidelines for Children and Youth [25] which recommend 60 minutes of physical activity per day for children 11–17. Because several studies have found the relationship between physical activity and academic achievement to have an inverse-U shape rather than a positive dose-response shape [26,27], we divided the days per week achieving 60 minutes of physical activity into three categories: 0–2 days, 3–5 days, and 6–7 days.

### Dietary aspects

Diet was assessed using a short food frequency questionnaire [28,29] and several free-standing questions about dietary habits. In order to reduce the number of variables and identify essential groupings of data from the short food frequency questionnaire, we conducted exploratory factor analysis with oblique rotation to allow for correlation between factors. This method was used to identify foods and behaviours from the diet-related questions that frequently occur together, such as children reporting frequently eating ‘vegetables’ being also more likely to frequently eat ‘orange vegetables (carrots, squash, sweet potato, etc.),’ which are two separate items in the questionnaire. The factor scores that were generated were used in regression analyses to quantify each factor’s association with academic achievement.

Our factor analysis of diet-related variables identified three factors from 16 variables. We named these: (1) Junk Food, (2) Vegetables, Pulses, and Fruit, and (3) Healthy Eating Habits. The Healthy Eating Habits factor comprised of responses to questions about the frequency of eating breakfast and consuming meals in the presence of family. Although the food frequency questionnaire item ‘Game from hunting (moose, caribou, venison, etc.)’ was included in the factor analysis, it did not load onto any factor using the specified cutoff (0.4) and as such was not included. Table 1 lists all food frequency questionnaire items and their loadings onto respective factors. All factor loadings indicate that the higher the item is reported being consumed, the associated factor score increases. Conversely, if the item was reported to be consumed infrequently, the factor score decreases.

**Table 1:** Factor loadings of items from short food-frequency questionnaire onto determined dietary aspects.

Dietary Aspect	Factor Loadings*
Junk Food and Drinks	Sweets (candy or chocolate) (0.56), Coke or other soft drinks that contain sugar (0.74), Diet Coke or other diet soft drinks (0.48), Eating in a fast food restaurant (0.53), Potato chips (0.70), Energy drinks (Red Bull, Rock Star, Guru, etc.)(0.49), Sports drinks (Gatorade, Powerade, etc.)(0.49)
Vegetables, Pulses, and Fruit	Fruits (0.75), Vegetables (0.80), Orange vegetables (carrots, squash, sweet potato, etc.)(0.77), Fruit juice (0.39), Meat alternates (beans, lentils, tofu, eggs, peanut butter, etc.) (0.53)
Healthy Eating Habits	Eating breakfast on weekdays (0.69), Eating breakfast on the weekends (0.66), Eating breakfast with family members (0.74), Eating evening meals with family members (0.59)

*Higher factor loadings indicate higher contribution of the item to overall factor score.

### Sleep

Students reported their bedtime (“turned out the light and gone to sleep”) and wake-up time on school days and weekend days over the past week. Sleep duration was organized into categories of meeting and not meeting recommendations in reference to age group-specific thresholds in the Canadian 24-hour Movement Guidelines for Children and Youth [25]. Children between the ages of 8–13 are recommended to get between 9 and 11 hours of sleep per night, whereas youth between the ages of 14–17 are recommended to get 8 and 10 hours of sleep per night.

### Screen time

Average daily screen time was assessed using four questions about typical usage of various screens on weekdays and weekends. Students were asked for both a typical weekday and a typical weekend day: How many hours a day, in your free time, do you usually spend watching TV, videos (including YouTube or similar services), DVDs, and other entertainment on a screen? Possible responses were: ‘None at all’, ‘About half an hour a day’, ‘About one hour’, etc, until ‘About 7 or more hours a day.’ This question is also asked for ‘time spent playing games on a computer, console, tablet (like iPad), smartphone, or other electronic device (not including moving or fitness games)?, and ‘time spent using electronic devices such as computers, tablets (like iPad), or smartphones for other purposes (e.g., homework, emailing, tweeting, Facebook, chatting, surfing the internet)?’. Responses were totaled for all devices for weekdays and weekends; the average of these two totals was calculated to represent average daily screen time during a typical week. Categories were organized in reference to the Canadian 24-hour Movement Guidelines for Children and Youth [25] where <2 hours per day of screen time is recommended.

### Body weight status

Height and weight were self-reported by students in the unit of their choice. Reponses for height were converted to centimeters if reported in inches and responses for weight were converted to kilograms if reported in pounds. These values were used to calculate body mass index (kg/m^2^), which became a measure of body weight status as per age- and sex- specific WHO Child Growth Standards [30]. Children were classified as being thin if they were more than 2 standard deviations below the mean and severely thin if they were more than 3 standard deviations below the mean. These two categories were collapsed into one because of small cell sizes. Children were classified as overweight if they were more than 1 standard deviation above the mean and obese if they were more than two standard deviations above the mean [30].

### Socioeconomic status

Socioeconomic status was determined using the Family Affluence Scale (FAS). The FAS is a validated scale that is used across all countries using the HBSC survey, and has been used in aggregate analyses focusing on the relationship between SES and adolescent health [31]. The FAS comprises of questions that ask about material goods (vehicles, individual bedrooms, and computers) and vacations to assess family wealth. The FAS generates a score between 0–9, with values of 0–2 corresponding with low affluence, 3–5 corresponding with medium affluence, and 6–9 corresponding with high affluence [31]. These categories were used in this analysis with low affluence as the reference category.

### Statistical analysis

Mixed-effects logistic regression was employed given that the proportional odds assumption required for ordinal logistic regression was violated. Bootstrapping techniques were employed to address the complex sampling design of the survey. As participating students are nested within school environments, while schools are nested within provinces, students are more likely to be similar to other students within their school environment, and in their province or territory, as education is under the jurisdiction of provinces and territories in Canada. As such, both schools and provinces were treated as levels of clustering within the sample. The ICCs for clustering at the school and provincial level, respectively, were 0.11 and 0.06 respectively. In order to determine if the fit of the clustered models were significantly better than a model that did not consider the clustering, likelihood ratio tests were performed. Results indicated that the clustered model was a statistically significantly better fit than one that ignored clustering (p<0.001). Odds ratios are interpreted as the likelihood of achieving at an Excellent level (Mostly A’s or Mostly A’s and B’s) compared to a ‘Fair’ level (Mostly B’c and C’s and below). Lifestyle behaviours (physical activity, diet, sleep, and screen time) and confounders (age, sex, and SES) were first considered individually in a univariable analysis to assess unadjusted effects, and then were included together in a fully adjusted model.

## Results

Students with incomplete data who were excluded from analysis had significantly lower socioeconomic status, were more likely to have the recommended levels of physical activity, higher Junk Foods and Drinks scores, and more screen time than those with complete data. Table 2 describes the demographic characteristics of participating students within the HBSC survey who were included in the present analysis. Most students (76.1%) reported having Excellent grades. Most students (45.6%) reported getting 60 minutes of physical activity 3–5 days per week. Approximately two-thirds of children met recommendations for sleep (66.3%). Only 11.6% of students reported meeting recommendations for screen time (<2 hours per day), while 47.0% of students reported getting 7+ hours of screen time per day. Finally, the majority of students reported heights and weights that resulted in a normal body mass index (69.4%). Three percent of students were severely thin or thin, 18.9% were categorized as overweight, and 8.7% were obese.

**Table 2:** Descriptive statistics of participants in the 2014 health behaviour of School-Aged Children questionnaire in Canada (weighted estimates based on 28,608 observations).

**Sex, %**	
Girl	50.9
Boy	49.1
**Age, mean (range)**	14.1(8.7–18.4)
**Family Affluence Scale, %**	
Low Affluence (0–2 points)	2.2
Medium Affluence (3–5 points)	30.5
High Affluence (6–9 points)	67.3
**Grade, %**	
6	15.4
7	19.4
8	19.7
9	23.6
10	21.9
**Academic Achievement, %**	
Excellent	76.1
Fair	23.9
**Physical Activity (days per week active for at least 60 minutes), %**	
6–7 days	38.1
3–5 days	45.6
0–2 days	16.3
Diet Factor Score (mean = 0, SD* = 1), range	
Junk Foods and Drinks	-2.41, 5.67
Vegetables, Pulses, and Fruit	-3.60, 3.24
Healthy Eating Habits	-3.96, 1.91
**Sleep, %**	
Meeting Recommendations	66.3
Not Meeting Recommendations	33.7
**Screen Time, %**	
<2 hours	11.6
2–4 hours	18.0
4–7 hours	23.5
>7 hours	47.0
**Body Mass Index Group**	
Normal	69.4
Severely Thin or Thin	3.0
Overweight	18.9
Obese	8.7

*SD = standard deviation

Table 3 shows the associations of physical activity, dietary factor scores, sleep, screen time, and body mass index with academic achievement. The univariable column represents unadjusted odds ratios between individual factors and likelihood of achieving Excellent grades. Fewer days where adequate physical activity was achieved, increasing junk food score, not meeting sleep recommendations, a high number of hours a day spent using screens, and being overweight or obese were negatively associated with likelihood of achieving a higher academic grading level. Youth who reported achieving 60 minutes of physical activity for 0–2 days per week had half the odds of achieving Excellent grades compared to children who had the recommended 60 minutes of physical activity 6–7 days per week (Table 3, Univariable Model, OR: 0.49: 95%CI [0.45, 0.54]). Children who reported 7+ hours of screen time per day had 40% reduced odds of achieving Excellent grades compared to those who met the recommended <2 hours per day (Table 3, Univariable Model: OR: 0.60 [0.54, 0.67]). Having either too short or too long of a sleep duration compared to the recommended hours per night was associated with 0.67 times the odds of achieving Excellent grades (Table 3, Univariable Model: OR: 0.67 [95%CI: 0.62, 0.72]). Higher consumption of Vegetables, Pulses, and Fruits higher scores in Healthy Eating Habits and moderate levels of screen time were positively associated with achieving Excellent grades. Every one unit increase in Healthy Eating Habits score resulting in 1.37 times the odds of achieving at a level of Excellent. Students who reported 2–4 hours per day of screen time had 1.23 times the odds of achieving Excellent grades compared to students who met the recommended amount of screen time of <2 hours per day (Table 3, Multivariable Model: OR: 1.23 [95%CI: 1.08, 1.41]).

**Table 3:** Results of multi-level ordinal logistic regression of the association of lifestyle behaviours with academic achievement among 11–15 year old Canadians in 2014.

	Univariable	Multivariable**
	Odds Ratios* (95% CI)	Odds Ratios* (95% CI)
**Physical Activity (days per week active for at least 60 minutes)**		
6–7 days (reference)	1.00	1.00
3–5 days	**0.77 (0.72, 0.84)**	**0.89 (0.80, 0.99)**
0–2 days	**0.49 (0.45, 0.54)**	**0.65 (0.54, 0.77)**
**Diet Factor**		
Junk Foods and Drinks	**0.72 (0.70, 0.74)**	**0.74 (0.70, 0.77)**
Vegetables, Pulses, and Fruit	**1.37 (1.33, 1.42)**	**1.22 (1.17, 1.28)**
Healthy Eating Habits	**1.71 (1.66, 1.77)**	**1.55 (1.48, 1.63)**
**Sleep**		
Adequate (reference)	1.00	1.00
Not recommended	**0.67 (0.62, 0.72)**	**0.85 (0.77, 0.93)**
**Screen Time**		
<2 hours (reference)	1.00	1.00
2–4 hours	**1.23 (1.08, 1.41)**	**1.24 (1.00, 1.53)**
4–7 hours	1.02 (0.88, 1.19)	1.25 (1.00, 1.50)
>7 hours	**0.60, 0.54, 0.67)**	0.89 (0.73, 1.09)
**BMI Group**		
Normal (reference)	1.00	1.00
Severely Thin or Thin	1.04 (0.82, 1.32)	1.14 (0.88, 1.48)
Overweight	**0.74 (0.66, 0.83)**	**0.86 (0.77, 0.95)**
Obese	**0.56 (0.51, 0.62)**	**0.68 (0.59, 0.79)**

*Odds Ratios for logistic regression. Odds ratios above 1 represent increased odds of achieving at a level of Excellent, while odds ratios below one represent decreased odds of achieving at a level of Excellent.

**Multivariable model adjusted for all variables listed here as well as student age, sex, and socioeconomic status.

The multivariable results in Table 3 represent odds ratios that are fully adjusted for all variables of interest and potential confounders considered in the analysis. The negative association between fewer days of physical activity and academic achievement continued, although the effects were slightly attenuated. Compared to youth who reported getting 60 minutes per day of physical activity 6 or 7 days a week, youth who reported getting 3–5 days or 0–2 days had 0.89 and 0.65 times the odds of achieving Excellent grades, respectively (Table 3, Multivariable Model: OR: 0.89, [95%CI 0.80, 0.99], and OR: 0.65, [95%CI 0.56, 0.77]). Associations between dietary aspects and academic achievement remained consistent with univariable results. For every unit increase in score for Junk Foods and Drinks, students had 0.74 times the odds of achieving Excellent grades (Table 3, Multivariable Model: OR: 0.74 [95% CI: 0.70, 0.77]). For every unit increase in Vegetables, Pulses, and Fruit score and Healthy Eating Habits score, students had 1.22 and 1.55 times the odds of achieving Excellent grades, respectively. Students with inadequate sleep duration had 0.85 times the odds of achieving Excellent grades compared to those with adequate sleep duration (Table 3, Multivariable Model: OR: 0.85 [0.77, 0.93]). Significant univariable associations between screen time and academic achievement were completely attenuated following adjustment for other health behaviours and confounders.

## Discussion

In early adolescents from Canada, we found that all of physical activity, diet, sleep, and screen time had independent effects on academic achievement. We observed that increased consumption of vegetables, pulses, and fruits, and more regular healthy eating habits, were positively associated with higher academic achievement. Fewer days of achieving adequate physical activity, increased consumption of junk foods and drinks, not meeting recommendations for sleep duration, and overweight and obesity were negatively associated with higher academic achievement. This is the largest study to date, of the few that have been conducted, to consider all of these predictors in their independent relationships with academic achievement. The findings about the independent associations of lifestyle behaviours with academic achievement are consistent with the limited studies that have previously investigated this objective.

Students who had fewer days of achieving the recommended amounts of physical activity in a week had decreased likelihood of achieving Excellent grades. Students achieving the fewest days of adequate physical activity had 33% reduced odds of achieving Excellent grades compared to students achieving the highest number of days. Studies investigating the importance of physical activity and physical fitness for academic achievement have been predominantly indicative of a positive relationship between higher levels of physical activity and academic achievement, although there have been some inconsistencies [32–34]. This study supports previous findings that suggest a positive, linear relationship between physical activity and academic achievement [34–36]. The positive influence of physical activity on academic achievement, independent of other lifestyle behaviours, is consistent with findings from Ickovics et al (2014), Martinez-Gomez et al (2012), and Vassiloudis et al (2014), but inconsistent with those of our team’s previous work in Nova Scotia, Canada, which found a null relationship between physical activity and academic achievement once other lifestyle behaviours were considered [19–22,37].

The findings from the present study about diet are largely congruent with previous findings about the relationship between diet and academic achievement. Consumption of vegetables and fruit has consistently had a positive association with academic achievement [38, 39] and junk foods, including beverages and snacks high in sugar and fat and frequent consumption of fast foods, have a similar negative association with academic achievement [39,40]. In addition, breakfast consumption has been highly studied in its relationship with academic achievement; the present findings about Healthy Eating Habits, which comprise regular breakfast consumption, are consistent with the positive findings of previous work [41,42]. However, although regular consumption of meals with the family has been shown to be beneficial for children’s diet and reduce likelihood of overweight and obesity [43,44], few studies have considered this in relation to academic achievement. In the present study, of all measures of diet included in this study (Vegetables, Pulses, and Fruit, Healthy Eating Habits, and Junk Foods and Beverages), Healthy Eating Habits, which include regularly consuming breakfast and dinner, and doing so in the presence of family, had the strongest positive relationship (50% increased odds with more frequent healthy habits) with academic achievement when considered simultaneously with other aspects of the diet, similar to findings from Ickovics et al (2014) who also looked at diet, physical activity, sleep, screen time and body weight status with academic achievement in a sample of urban American youth [20]. Frequently eating meals in the presence of family is associated with better psychosocial well-being among children which may be a contributing factor to better academic success [45].

Not meeting recommendations for sleep (representing having a sleep duration that is either too short or too long compared to recommendations) was negatively associated with academic achievement in youth. These findings are consistent with previous literature [46]. Because increased screen time has been shown to negatively affect sleep as well as academic achievement [47], it is possible that negative relationships between poor sleep and academic achievement are actually attributed to increased screen time [22]. However, the present findings demonstrate an independent, negative association of sleep with academic achievement, while screen time was found to have no association with academic achievement following adjustment for sleep and other. The lack of association of high levels of screen time with academic achievement is inconsistent with the conclusions recent systematic reviews, in particular for observational studies [9]. However, it has been acknowledged that self-reported screen time is of lower validity for the measurement of screen time or sedentary behavior, and though the majority of existing results indicate a positive association, there is still substantial inconsistency among cross-sectional studies and very few longitudinal studies using objective measures [9]. Future studies using objective measurements of sedentary behavior, taking into consideration sleep habits, and longitudinal analyses would benefit this area of the literature and clarify this relationship.

Overweight and obesity had strong, significant relationships with academic achievement independent of lifestyle behaviours. Although there are many studies investigating the relationship between body weight status and academic achievement, there are few that have taken the lifestyle behaviours that contribute to both weight status and academic achievement into account [12]. Of the existing studies [19–21,37] three of the four have found that overweight or obesity no longer had an association with academic achievement following the inclusion of lifestyle behaviours into an associative model. The results from the present study are inconsistent with the findings of these three studies and consistent with the fourth that did find an association. The present study used self-reported measures of academic achievement and body weight status, the work of Martinez-Gomez et al. (2012) used self-reported grades and measured body weight status [19], and the work of Vassiloudis et al (2014) used teacher-assessed grades and measured body weight status [21]. The studies by Wang et al. (2008) and Faught et al. (2017) used both objectively measured academic achievement and body weight status [22,37]. The present study categorized body weight status using the WHO Growth Reference standards [30], while the other four studies used the International Obesity Task Force (IOTF) reference standards [48]. The distinction between these two methods is significant–the WHO Growth Reference standards are generated in reference to an international cohort raised in strictly controlled, idealistic conditions, while the IOTF is meant to be representative of how an international cohort of children and youth grow under average circumstances [31,48,49]. Using a nationally representative sample of Canadian children and youth, Shields et al (2010) calculated childhood obesity rates using both IOTF and WHO reference cutoffs, and found that the WHO estimates of overweight and obesity were consistently and substantially higher than IOTF estimates. For boys 6–11, estimates of overweight including obesity using the WHO references were 14.0% higher than estimates using the IOTF cutpoints [49]. Results from the comparisons conducted by Shields et al and those from the present study are indicative that the use of different cutpoints across studies of the same objective may contribute to different results. The study by Vassiloudis et al (2014) was comprised of a sample of students with a much higher prevalence of obesity than those considered by the other students that was more comparable with the findings of the present study [21]. Consideration must be taken in deciding which cutpoints to employ, as well as prevalence of obesity, and how these may affect results and comparisons with existing literature. Regardless, the health promotion messages remain the same: the academic achievement of students of any body weight status will benefit from more frequent physical activity, a better diet, adequate sleep, and reduced screen time.

The present study’s strengths include a large, representative sample of Canadian children and youth with extensive data on their lifestyle behaviours, academic achievement, and socioeconomic status. Although statistical significance for small effect sizes can be found in studies using large datasets, the findings from this thesis provide compelling evidence of a strong relationship between lifestyle behaviours and academic achievement. However, this study is cross-sectional in nature so no statement of causality can be made from these results. In addition, there is potential for health behaviours to moderate the association between socioeconomic status and academic achievement. Further studies of this objective using longitudinal datasets are required to provide stronger evidence about relationships rather than associations, and to assess mediating and moderating effects. While measures are validated, the questions are brief to reduce participant burden and do not provide the depth of information that more intensive measurement would provide for each item. In addition, the collection of health and academic achievement data simultaneously may results in some differential bias as both health and academic achievement data may be overestimated by adolescents.

The present study contributes to the literature investigating the relationship among lifestyle behaviours, body weight status, and academic achievement. Findings from this study, and in the context of other studies with a similar objective, indicate that healthier lifestyle behaviours, regardless of body weight status, SES, and gender, contribute to better academic achievement. As such, equitable, accessible interventions to improve lifestyle behaviours to improve academic achievement and reduce the likelihood of adverse subsequent health outcomes are needed. The Ottawa Charter for Health Promotion (1986) states that, “health is created and lived by people within the context of their everyday life; where they learn, work, play and love [50].” In light of the present study’s findings, health promoting interventions within children and youth’s key settings, like schools and family environments, present a potential opportunity to support both health and academic achievement among children and early adolescents. Future studies investigating these associations using objective measures and longitudinal approaches would be valuable to better understand the potential causal relationship between health behaviours and academic achievement as well as investigate potential mediation and moderation of relationships between various factors and academic achievement.
